# Proteomic analysis of the processes leading to *Madurella mycetomatis* grain formation in *Galleria mellonella* larvae

**DOI:** 10.1371/journal.pntd.0008190

**Published:** 2020-04-08

**Authors:** Gerard Sheehan, Mickey Konings, Wilson Lim, Ahmed Fahal, Kevin Kavanagh, Wendy W. J. van de Sande

**Affiliations:** 1 Medical Mycology Laboratory, Department of Biology, Maynooth University, Co. Kildare, Ireland; 2 Department of Medical Microbiology and Infectious Diseases, Erasmus MC, University Medical Centre Rotterdam, Rotterdam, The Netherlands; 3 Mycetoma Research Centre, Khartoum, Sudan; Rutgers University, UNITED STATES

## Abstract

Mycetoma is a neglected chronic and granulomatous infection primarily associated with the fungal pathogen *Madurella mycetomatis*. Characteristic of this infection is the formation of grains. However, the processes leading to grain formation are not known. In this study, we employed a proteomic approach to characterise *M*. *mycetomatis* grain formation in *Galleria mellonella* larvae and map the processes leading to grain formation over time. For this, at 1 day, 3 days and 7 days post-inoculation, proteins from grains and hemolymph were extracted and analysed by label-free mass spectrometry. A total of 87, 51 and 48 *M*. *mycetomatis* proteins and 713, 997, 18 *G*. *mellonella* proteins were found in grains on day 1, 3 and 7 post-inoculation respectively. *M*. *mycetomatis* proteins were mainly involved in cellular metabolic processes and numerous enzymes were encountered. *G*. *mellonella* proteins were primarily involved in the nodulation process. The proteins identified were linked to nodulation and grain formation and four steps of grain formation were identified. The results of this proteomic approach could in the future be used to design novel strategies to interfere with mycetoma grain formation and to combat this difficult to treat infection.

## Author summary

Although grain formation is the hallmark of mycetoma, so far the pathways leading to grain formation were not studied. Since our hypothesis is that both host and pathogen play a role in this process, we aimed to study this process in a model system. Grains can be formed in the invertebrate *Galleria mellonella* and different stages of grain formation can be noted within the larvae. We therefore infected *G*. *mellonella* with the mycetoma causative agent *Madurella mycetomatis*, and monitored grain formation over time. At day 1, day 3 and day 7 post-inoculation, grains and hemolymph were obtained from infected larvae. Proteins were isolated and identified by label-free mass spectrometry. By analyzing the proteins found in both host and pathogen on the different time points, we were able to develop a grain model over time. This grain model can in the future be used to identify novel treatments for this difficult to treat infection.

## Introduction

*Madurella mycetomatis* is the dominant causative agent of eumycetoma, a chronic granulomatous type infection which is severely debilitating to its sufferers due to tissue destructions[1]. Mycetoma is endemic in tropical and subtropical regions but the highest prevalence is documented within the African continent [2]. The disease is of gradual onset, and the symptoms may take years to develop. Mycetoma is associated with large subcutaneous swellings on the extremities which hinder the patients in their daily activities and the formation of sinuses that discharge grains [3]. These grains are considered the key feature of mycetoma and are thought to be formed as a defence mechanism by the fungus against the host immune system [4]. Although it is currently not known how these grains are formed, we do know that grains consist of melanin, chitin, lipids and proteins [5–9]. Chitin and melanin are present as thick layers on hyphal walls [6, 7] and proteins were found both on the cells as well as in the cement material [8, 9] surrounding the hyphae. Furthermore, zinc, copper and calcium concentrations were significantly higher in *M*. *mycetomatis* infected tissues than in control tissue which could be contributed to the formation of the grain cement matrix [10]. Grains are only found *in vivo*, and animal models are needed to produce these grains [11–13].

Recently, we demonstrated that grains can also be formed in the invertebrate *Galleria mellonella* [11]. The invertebrate *G*. *mellonella* is a recognized *in vivo* system to assess the virulence of fungal species, assess the toxicity and efficacy of novel anti-fungal drugs and more recently to study the response of the innate immune response towards an invading fungal pathogen [14–20]. The wide spread acceptance of this model is due in part to the lack of legal and ethical considerations associated with larvae, their ease of use (inoculation, low cost, ability to generate results within 24–48 hours) and the fact that results correlate closely with those obtained using mice [21]. This is due to the similarities between the insect immune system and the mammalian innate immune response. Insect hemocytes show many similarities (e.g., phagocytosis, superoxide production) to mammalian phagocytes [22] and many of the receptors (e.g., Toll) and response pathways (e.g., coagulation and melanisation) in insects are comparable to those in mammals [23–25]. Furthermore, these larvae produce a plethora of antimicrobial peptides, which are similar to their equivalents in mammals in response to invasion by human pathogens [26–29]. Next to *M*. *mycetomatis*, larvae have been exploited to study the virulence of a range of fungal pathogens including *Candida albicans* [15], *Cryptococcus* neoformans [30], *Candida auris* [31], *Aspergillus flavus* [32], *A*. *fumigatus* [16, 17], *Fusarium oxysporum* [33], *Paracoccidioides lutzii* and *Histoplasma capsulatum* [34].

The grains formed in *G*. *mellonella* are similar to those extracted from human and mammalian biopsies [11]. Also the immune reaction surrounding the grain demonstrated some similarities. The hemocytes around the *M*. *mycetomtatis* grain within *G*. *mellonella* larvae are similar to neutrophils surrounding the grains in human. Hemocytes and neutrophils share similar receptors and transcription factors and both degranulate, form reactive oxygen species and extracellular nets [22]. However, unlike in human, in the *G*. *mellonella* grain model, grain formation can be followed over time and different grain developmental stages can be noted [11]. These developmental stages also resembled the developmental stages found in murine grains [9]. Furthermore, in both *G*. *mellonella* larvae and in mice, similar responses to antifungal agents were noted. In mice, grain formation could be prevented by administering amphotericin B but not by itraconazole [35], while in larvae prolonged survival was noted with amphotericin B, but not with itraconazole [36]. This indicates that the *M*. *mycetomatis* grain model in *G*. *mellonella* larvae could be a suitable model to unravel the processes leading to grain formation. This information can be useful to understand the pathology of mycetoma and to identify novel preventative and therapeutic measurements against mycetoma.

We therefore used the *G*. *mellonella* grain model to profile the changes of larval proteome following infection by *M*. *mycetomatis* and to identify proteins secreted by *M*. *mycetomatis* during grain formation in order to understand the biological processes involved in grain formation *in vivo*. This information will help us in the future to find compounds which specifically inhibit the processes leading to grain formation.

## Materials and methods

### Infection of *G*. *mellonella* larvae with *M*. *mycetomatis*

*G*. *mellonella* larvae were obtained from Terra Equipment Voedseldieren (Cuijk, The Netherlands) and kept at room temperature on wood shavings in the dark until use. Larvae of approximately 300–500 mg showing no discoloration were used within five days of receipt. Larvae were infected with *M*. *mycetomatis* genome strain mm55 [37] via the last left proleg. To prepare the inoculum for the *G*. *mellonella* larvae, *M*. *mycetomatis* mycelia obtained from Sabouraud Dextrose plates were sonicated for 30 s at 28 micron (Soniprep, Beun de Ronde, The Netherlands) and added to 500 ml colorless RPMI 1640 medium supplemented with L-glutamine (0.3 g/liter), 20 mM mopholinepropanesulfonic acid (MOPS) and chloramphenicol (100 mg/liter; Oxoid, Basingstoke, United Kingdom). After two weeks incubation at 37°C, the mycelia were separated and washed by vacuum filtration (Nalgene, Abcoude, The Netherlands). Wet weights of the mycelia were determined and a suspension containing 100 mg wet weight per ml in phosphate-buffered saline (PBS) was sonicated for 2 min at 28 micron. The resulting homogenous suspension was washed once in PBS and diluted to a final inoculum size of 4 mg wet weight per 40 μl PBS corresponding to 600–850 CFU/larvae. Inoculation was performed by injecting 40 μl of the fungal suspension in the last left pro-leg with an insulin 29G U-100 needle (BD diagnostics, Sparsk, USA). To monitor the course of infection, in a separate group consisting of 15 larvae, survival was recorded on a daily basis for ten days. Pupa formed during these then days were left out of the equation. In all experiments, non-infected larvae were used as control groups.

### Burden of infection

At day 1, day 3 and day 7 after inoculation hemolymph and grains were collected from five larvae per time point. At the same time points an additional five larvae were fixed in 10% buffered formalin to determine the burden of infection. Since the larval exoskeleton is impenetrable to most fixative reagents, 100 μl of the 10% buffered formalin was injected into the larvae [11]. After 24 h fixation, whole larvae were dissected longitudinally into two halves with a scalpel and fixed in 10% buffered formalin for at least another 48 h. The two halves were processed for histology. Sections were stained with hematoxylin and eosin (HE) and Grocott methenamine silver. To assess the number of grains per larvae, the grains were manually counted under a light microscope mounted with a Canon EOS70D camera (Canon Inc.) by two independent scientists. Grains were magnified 40x and visualized on the computer screen using the supplied EOS Utility software (Canon Inc.) and categorized into large, medium or small sizes using the enlargement display frame present in the Live View Shooting mode. Under 40x magnification, the enlargement display frame has a width and height of approximately 250 μm and 160 μm and sums up to a dimension of 0.04 mm^2^. Grains that were larger than half of the display frame were categorized as large (*>*0.02 mm^2^). Grains that were larger than a quarter of the frame but smaller than half of the frame are categorized as medium (0.01 ± 0.019 mm^2^) and those between one-eighth to a quarter of the display frame (0.005 ± 0.009 mm^2^) were categorized as small. The sum of all large, medium and small grains present in larvae was used to represent the total number of grains in the larvae. To determine the total size of grains in the larvae, the sum of all grains in a larva multiplied by the minimum size of their respective category (large: 0.02 mm^2^, medium: 0.01 mm^2^ and small: 0.005 mm^2^) was used.

### Proteomic response of *G*. *mellonella* larval hemolymph to *M*. *mycetomatis*

To determine the proteins present in the larval hemolymph of *M*. *mycetomatis* infected larvae, hemocytes were removed by centrifugation at 10,000 rcf for 10 minutes to obtain cell free hemolymph. The cell-free hemolymph was diluted in PBS and the proteins were quantified by the Bradford protein assay. The proteins were then acetone precipitated (75 μg) overnight by the addition of 3 times the total volume of ice-cold acetone and subjected to label-free quantitative LC-MS/MS.

### Analysis of the *M*. *mycetomatis* grain proteome over time in *G*. *mellonella* larvae

*M*. *mycetomatis* grains were dissected from *G*. *mellonella* larvae, washed by centrifugation with PBS and frozen in PBS (volume = 100 μl) overnight. Grains were washed twice with PBS and resuspended in lysis buffer (7M Urea, 2M thiourea, 0.1M Tris-HCl supplemented with protease inhibitors [1 μg/ ml TLCK, Aprotinin, PMSF, leupeptin]), subjected to sonication (three cycles of 6 x 10 seconds pulses at 20% power) and clarified by centrifugation (10,000 x g for five minutes). Protein supernatant concentration was determined by Bradford protein assay and protein was acetone principiated (75 μg) overnight by the addition of three times total volume of ice-cold acetone. Proteins were subjected to label-free quantitative LC-MS/MS.

### Label-free proteomics workflow

Proteins were analyzed by using label-free quantitative LC-MS/MS by standardised protein purification procedures as described [20]. In summary, 0.75 μg of peptide mix was eluted onto a Q-Exactive (ThermoFisher Scientific, U.S.A) high resolution accurate mass spectrometer connected to a Dionex Ultimate 3000 (RSLCnano) chromatography system. Peptides were separated by an increasing acetonitrile gradient on a Biobasic C18 Picofrit column using a 65 min reverse-phase gradient at a flow rate of 250 nL /min. A high-resolution MS scan (300–2000 Dalton) was performed using the Orbitrap to select the 15 most intense ions prior to MS/MS.

Protein identification from the MS/MS data was performed using the Andromeda search engine in MaxQuant (version 1.2.2.5; http://maxquant.org/) to correlate the data against the proteome of *M*. *mycetomatis* (proteins in hemolymph and grains) obtained from Uniport and the EST contigs of *G*. *mellonella* (hemolymph and grain proteins) obtained in house.

Results processing, statistical analyses and graphics generation were conducted using Perseus v. 1.5.5.3 as described [20]. Proteins that had non-existent values (indicative of absence or very low abundance in a sample) were also included in statistical analysis of the total differentially expressed group following imputation of the zero values using a number close to the lowest value of the range of proteins plus or minus the standard deviation. After data imputation these proteins were also included in subsequent statistical analysis. The Search Tool for the Retrieval of INteracting Genes/Proteins (STRING) [38] v10.5 (http://string-db.org/) was used to map known and predicted protein:protein interactions. UniProt gene lists (extracted from Perseus) were inputted and analysed in STRING using the medium confidence (0.5) setting to produce interactive protein networks for proteins that increased and decreased in abundance.

### Statistical analysis

The difference in the number of grains or the size of the grains per time point were determined using the Mann-Whitney *U* test. A p-value of ≤ 0.05 was deemed significant.

## Results

### *M*. *mycetomatis* infection in *G*. *mellonella* larvae

**Fig 1:**
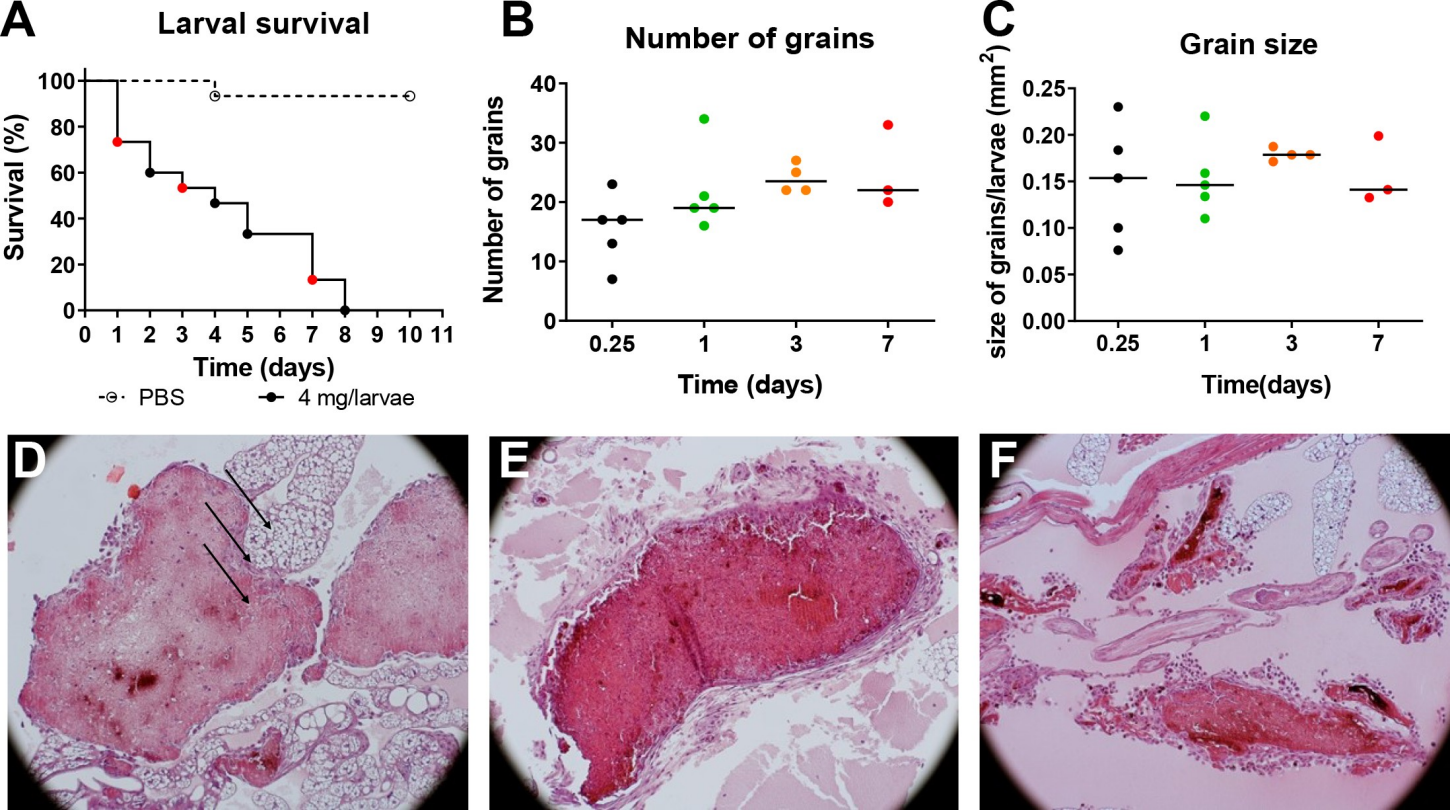
*M*. *mycetomatis* infection in *G*. *mellonella* larvae over time. A: Larval survival of PBS infected (---) and *M*. *mycetomatis* infected (------) larvae over 10 days, each day is represented with a dot. B: The number of *M*. *mycetomatis* grains present in the infected *G*. *mellonella* larvae at day 1, 3, and 7 after fungal inoculation as assessed by histology. C: The size of the *M*. *mycetomatis* grains present in the infected *G*. *mellonella* larvae at day 1, 3, and 7 after fungal inoculation as assessed by histology. D: Hematoxylin Eosin (HE) staining of a *M*. *mycetomatis* grain in a *G*. *mellonella* larvae, 1 day after fungal inoculation. Arrows, indicate the presence of hemocyte within the cement material of the grain. E: HE staining of a *M*. *mycetomatis* grain in a *G*. *mellonella* larvae, 3 days after fungal inoculation. F: HE staining of a *M*. *mycetomatis* grain in a *G*. *mellonella* larvae, 7 days after fungal inoculation.

As described before, when *G*. *mellonella* larvae were infected with *M*. *mycetomatis* a rapid decrease in larval survival was noted (**Fig 1A**)[11]. All larvae infected with 4 mg *M*. *mycetomatis* hyphal suspension per larvae died within eight days of infection (**Fig 1A**). During the course of infection, the number and the size of the grains within the larva remained constant (**Fig 1B and 1C**). No statistical significant differences were noted when day 1 after inoculation was compared to days 3 or 7 after inoculation (Mann-Whitney, p>0.05). However, the morphology of the grains did differ per time point. At day 1, cement material was present within the grain and individual hemocytes were trapped within this cement material. Hyphal cells were also clearly seen. At this time point, encapsulation was not noted. At 3 days after inoculation, the cement material was fully formed and no individual hemocytes were found inside the grains (**Fig 1E**). Some hemocytes were seen surrounding the grain. Furthermore, the grain became encapsulated. At 7 days after inoculation most larvae had died (**Fig 1A**). The surviving larvae showed grains with many hemocytes surrounding them (**Fig 1F**). The capsule surrounding the grain was also less prominent and it often had started to degrade.

### Grain formation over time

To determine which processes were involved in grain formation, the proteome of *M*. *mycetomatis* infected larvae at 1 day, 3 days and 7 days after inoculation was determined.

**Fig 2:**
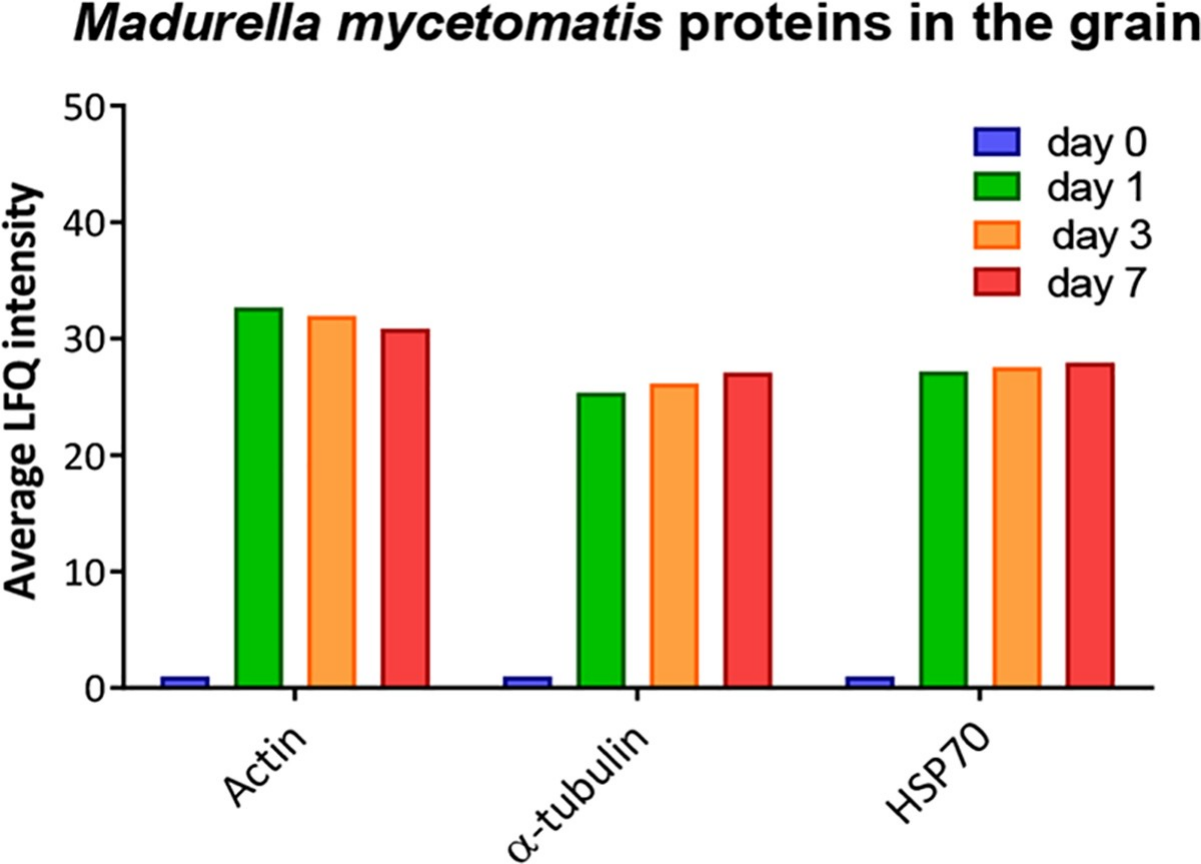
*M*. *mycetomatis* proteins in the grain. The average LFQ intensity of *M*. *mycetomatis* actin, alpha-tubulin and HSP70 obtained from grain samples at day 0, day 1, day 3 and day 7 after fungal inoculation.

**Fig 3:**
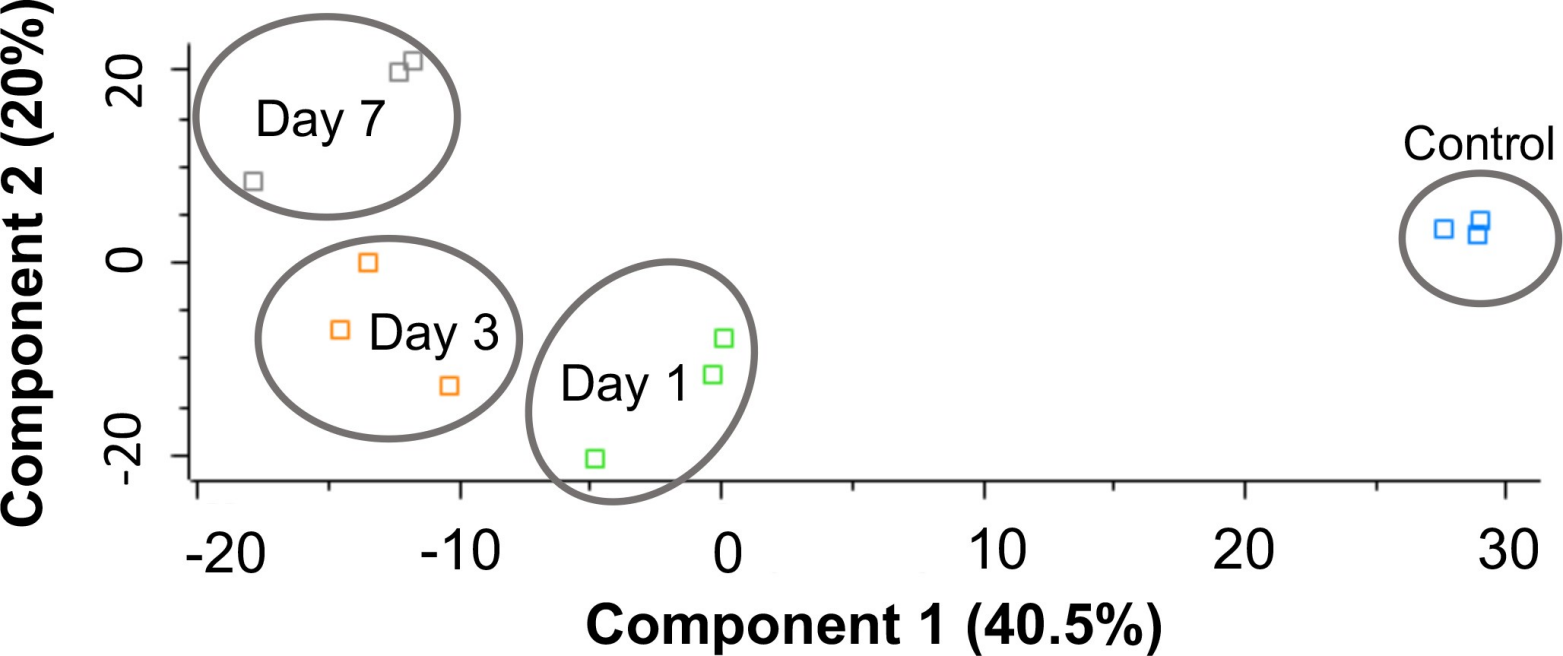
Shotgun quantitative proteomic analysis of hemolymph proteome following infection with *M*. *mycetomatis*. Principal component analysis (PCA) of control larval hemolymph proteome (0 hour) and infected larval hemolymph proteomes following infection with *M*. *mycetomatis* after infection for 24 hour, 72 hour and 7 days with a clear distinction between control and infected larvae.

Over time, different *M*. *mycetomatis* proteins were found to be expressed in the grain. The number of *M*. *mycetomatis* proteins inside the grain remained relatively stable over time, with 87 *M*. *mycetomatis* proteins identified at 1 day after inoculation, 51 at 3 days after inoculation and 48 found 7 days after inoculation. However, the nature of these proteins differed. From the proteins identified, only 22 *M*. *mycetomatis* proteins were present on all three time points tested **(Table 1 and S1 Table)**. These included household proteins such as actin, alpha-tubulin, histones, ribosomal proteins and the Woronin body **(Table 1).** Within these first 7 days, the grain appeared to remain metabolic active as on all time points enolase, ATP synthase and malate dehydrogenase were identified. This indicated that both glycolysis and the Kreb’s cycle were functioning. Stress response related proteins such as heat shock protein 60 (Hsp60) and heat shock protein 70 (Hsp70) were also found. Some of the *M*. *mycetomatis* proteins were not only contained in the grain but also found to be secreted in hemolymph. Of these proteins three were found to be secreted in all time points, two only at 3 and 7 days and the other 75 only in a single time point. The three *M*. *mycetomatis* proteins found in hemolymph at all three time-points were actin, alpha-tubulin and Hsp70 **(Fig 2)**. The two proteins found to be excreted only at 3 and 7 days were histone H4 and GTP-binding protein ypt1. Also the *G*. *mellonella* proteome in hemolymph differed in time **(Fig 3).** The proteome of infected larvae was clearly different to that from non-infected larvae.

**Table 1:** 22 *M*. *mycetomatis* proteins found in grain on all time points.

Protein	Protein ID
Actin	A0A175W1E5
Alpha-Tubulin chain	A0A175W8P0
Heat shock protein 70	A0A175WDC7
Histone H4	A0A175VRA2
Histone H2A	A0A175VTM6
Malate dehydrogenase	A0A175VPT8
Uncharacterized protein	A0A175VXZ6
Uncharacterized protein	A0A175WCN7
Heat shock protein 60	A0A175WCI9
ATP synthase subunit alpha	A0A175VRU2
Heat shock protein 70	A0A175WA11
Heat shock protein 90	A0A175VT02
Mitochondrial outer membrane protein porin	A0A175VWW7
GTP-binding protein ypt1	A0A175WGS8
Cell division control protein 48	A0A175VYV0
Uncharacterised protein	A0A175W2C0
Ribosomal protein	A0A175VN17
Uncharacterized protein	A0A175VY99
Putative pyruidoxal 5—phosphate synthase subunit pdx-1	A0A175WCW2
Enolase	A0A175W3F4
Protein Ecm33	A0A175WE26
Woronin body major protein	A0A175VPL2

### Proteome at 24h after inoculation

#### Grains

**Fig 4:**
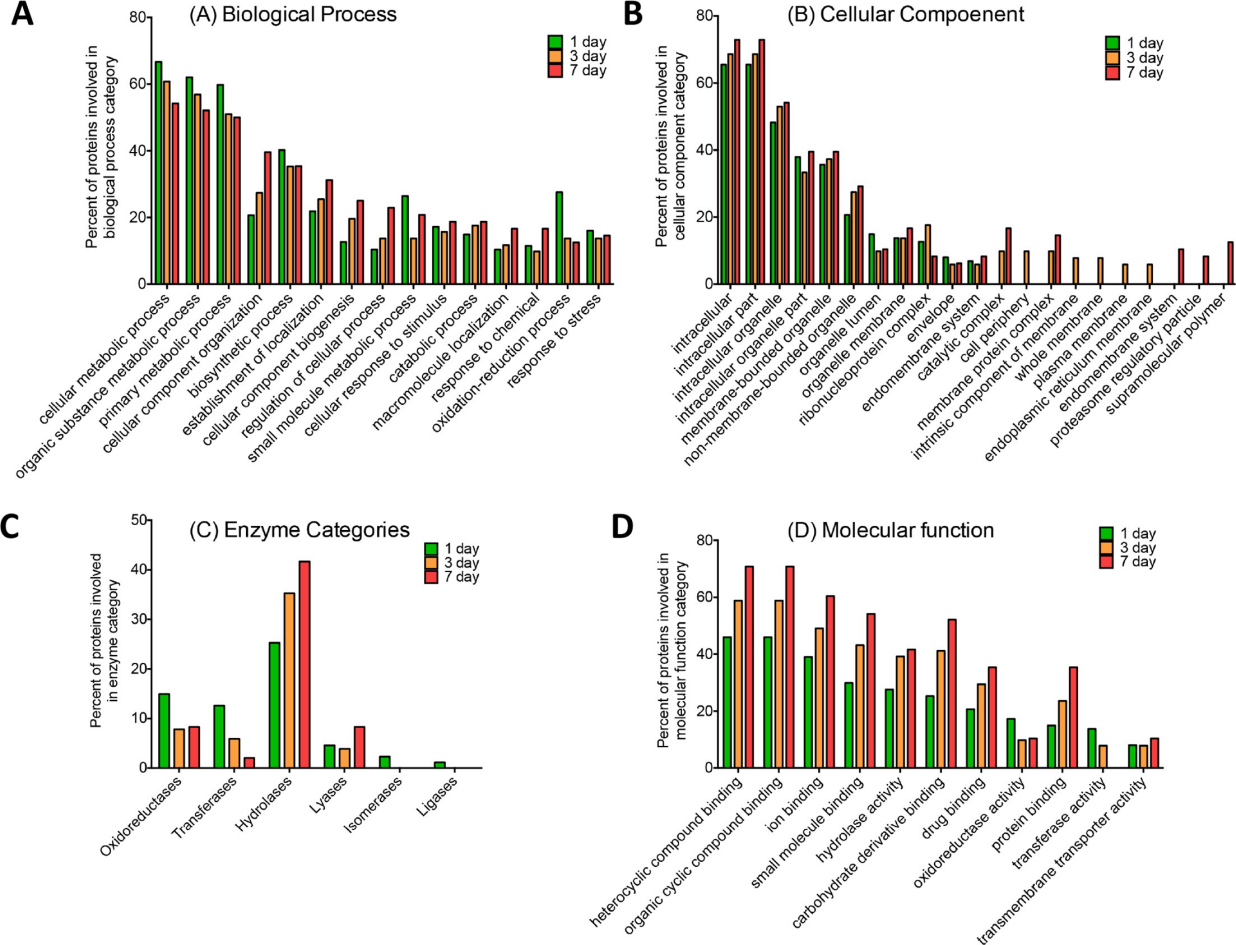
Bar chart showing changes in a number of proteins given various biological process (A), cellular component(B) [at level 3 ontology], enzyme categories (C) and molecular functions (D) on various time points. Proteins were assigned groups based on involvement in biological process, molecular functions and cellular component for the *M*. *mycetomatis* proteins identified within grain samples extracted from *G*. *mellonella* larvae during infection. Each group was assigned a percentage proportion of the total proteins found in the proteomic profile of each sample group.

At 24h after inoculation, a total of 87 *M*. *mycetomatis* proteins and 713 *G*. *mellonella* proteins were found within the *M*. *mycetomatis* grain. Among the 87 *M*. *mycetomatis* proteins identified, actin, Hsp70, malate dehydrogenase, heat shock protein 90 (Hsp90), mitochondrial outer membrane protein porin, enolase, protein Ecm33, elongation factor 2, histone H2A, ATP-dependent RNA helicase, histone H2B, nucleoside diphosphate kinase, superoxide dismutase, glyceraldehyde-3-phosphate dehydrogenase, phosphoglycerate kinase, citrate synthase, transaldolase, 6-phosphogluconate dehydrogenase, fructose-bisphosphate aldolase (Fba), elongation factor 1, transketolase and peptidyl-prolyl cis-trans isomerase (PPIase) corresponded to proteins also encountered in cell extracts of *Aspergillus fumigatus*, *Aspergillus flavus*, *Aspergillus terreurs*, *Aspergillus niger*, *Aspergillus nidulans*, *Coccidioides posadasii*, *Mucor circinelloides*, *Saccharomyces cerevisiae*, *Candida albicans*, *Candida tropicalis*, *Candida parapsilosis*, *Candida glabrata* and *Crytococcus neoformans* [39]. These proteins represent common fungal proteins **(S1 Table)**. A range of proteins associated with virulence (putative fungistatic metabolite, cyanovirin-N, phospholipase, enolase), nutrient acquisition from hemolymph (trehalose-phosphatase, trehalase), detoxification of the immune response (catalase-peroxidase, flavohemoprotein, superoxide dismutase), allergenic reactions (major allergen Asp f 2) and cell wall organization/repair (putative beta-glucosidase A, woronin body major protein) made up the vast majority of proteins within *M*. *mycetomatis* grains (**Fig 4A)**. Most of the proteins were predicted to be within the intracellular components of the cell **(Fig 4B)**. Hydrolases, oxidoreductases and transferases were the most enriched enzymes categories amongst proteins identified in grains produced by *M*. *mycetomatis* **(Fig 4C and 4D).** Proteins identified in the grain which were associated with the stress response were the 78 kDa glucose-regulated protein, ATP synthase subunit alpha, histone H2A, catalase-peroxidase, uncharacterized protein (A0A175VYV0), actin, flavohemoprotein, superoxide dismutase (A0A175W4W0, A0A175W7X9) and mitochondrial peroxiredoxin PRX1.

Out of the 713 *G*. *mellonella* proteins identified, 472 statistically significant differentially abundant (SSDA) proteins were present in the 1 day grain proteome relative to the 0 hour hemolymph proteome **(S2A and S2B Table).** *G*. *mellonella* proteins increased in grains at 1 day as compared to hemolymph control proteome were associated with the immune response (cecropin-D-like peptide (176 fold), gloverin (115 fold), 6tox (47 fold), lysozyme (18 fold), prophenol oxidase subunit 2 (12 fold), gloverin-like protein (11 fold) and macrophage migration inhibitory factor (7 fold)), protection against cellular stress (heat shock protein (hsp) (810 fold), hsp 90 (144 fold), thioredoxin (128 fold), prophenol oxidase activating enzyme 3 (47 fold), superoxide dismutase (116 fold) and glutathione-S-transferase-like protein (24 fold)), nodulation (hdd11 (147 fold), hdd1 (26 fold), hdd23 (21 fold), hemolin (19 fold), hdd1-like protein (19 fold), apolipoprotein D-like Protein (2 fold)) and a range of proteins that are primarily associated with intracellular processes (mitochondria, ribosome, proteasome) **(S2A Table).** *G*. *mellonella* proteins decreased at day 1 as compared to hemolymph control proteome were hexamerin (310 fold), arylphorin (13 fold), cationic peptide CP8 (5 fold), transferrin (5 fold) and also apolipophorins (4 fold) and lysozyme-like protein 1 (3 fold), **(S2B Table).**

#### Hemolymph

In the hemolymph, in total, 18 *M*. *mycetomatis* and 3217 *G*. *mellonella* peptides representing 330 proteins were identified. The most prominent *M*. *mycetomatis* proteins were Heat shock 70 kDa protein, Heat shock protein 90, Putative DNA helicase ino80, alpha-tubulin, Putative flavin-containing monooxygenase 1, Putative sterigmatocystin biosynthesis P450 monooxygenase stcF, Putative N-acetylglucosamine-6-phosphate deacetylase and TEL2-interacting protein 1 **(S3 Table)**.

**Fig 5:**
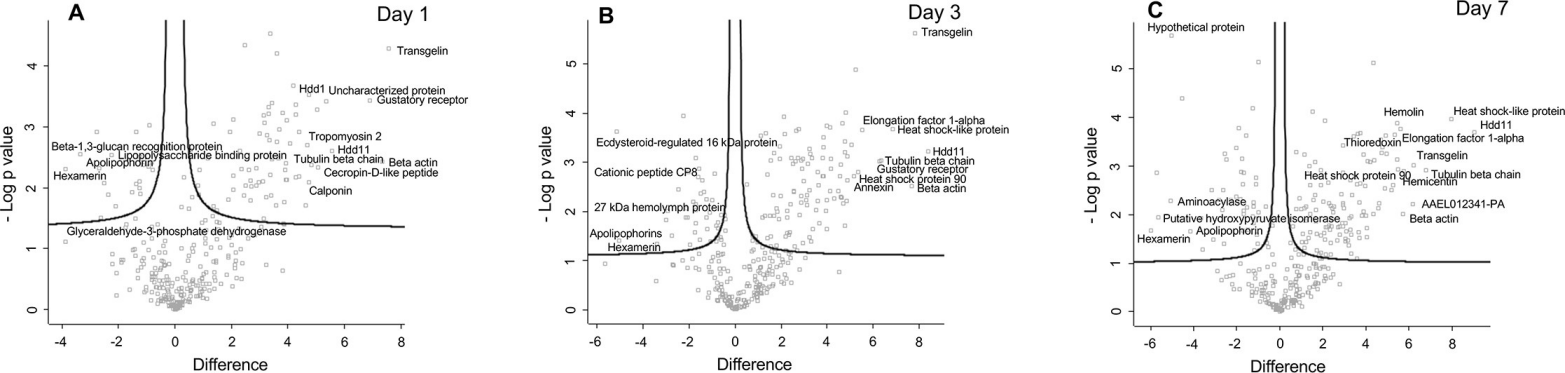
Proteomic responses of *G*. *mellonella* larvae following infection by *M*. *mycetomatis* mycelium after 24 hour (A), 72 hour (B) and 7 days (C) post infection. Volcano plots represent protein intensity difference (− log2 mean intensity difference) and significance in differences (− log P-value) based on a two-sided t-test. Proteins above the line are considered statistically significant (p value < 0.05) and those to the right and left of the vertical lines indicate relative fold changes > 1.5. Annotations are given for the most differentially abundant proteins identified in hemolymph from larvae infected with *M*. *mycetomatis* mycelium after 24 hour, 72 hour and 7 days. These plots are based upon post imputed data.

When the 330 *G*. *mellonella* proteins were compared to the proteome of uninfected *G*. *mellonella* proteins, 110 of the *G*. *mellonella* proteins were determined to be SSDA (ANOVA, p < 0.05) with a fold change of > 1.5 (Table **1**). *G*. *mellonella* proteins that were increased in abundance in larval hemolymph at day 1 were transgelin (191 fold), hdd11 (47 fold), cecropin-D-like peptide (33.5 fold), Hdd1 (28 fold), tropomyosin 2 (25 fold), thioredoxin (15.5 fold), hemicentin-like protein 1 (15 fold), prophenol oxidase activating enzyme 3 (11 fold), glutathione-s-transferase-like protein (10 fold), inhibitor of metalloproteinases [IMPI]; (9 fold) and gloverin (6 fold), (**Fig 5A, S4A Table)**. These proteins were subjected to GO analysis by Blast2GO software tool. A number of GO terms belonging to biological process (small molecule metabolic process, response to stress, cellular component organization and biosynthetic process), molecular function (ion binding, oxidoreductase activity, structural constituent of ribosome and organic cyclic compound binding), cellular component (intracellular organelle, membrane-bounded organelle, intracellular and endomembrane system) were significantly enriched within the dataset **(S5 Table)**. A number of proteins were decreased in abundance at day 1 as compared to day 0 hemolymph such as hexamerin (15 fold), beta-1,3-glucan recognition protein (10 fold), apolipophorin (6 fold), C-type lectin 21 precursor (4 fold) and anionic antimicrobial peptide 2 (2 fold) (**S4B Table)**.

### Proteome at 3 days after inoculation

#### Grains

In the grain proteome, a total of 51 *M*. *mycetomatis* proteins and 4746 peptides were detected representing 997 *G*. *mellonella* proteins. The *M*. *mycetomatis* proteins included cyanovirin-N, enolase, iron- sulfur cluster assembly protein, protein Ecm33, two-component system protein A, mitochondrial peroxiredoxin PRX1, heat shock protein 60, heat shock protein 90, heat shock 70 kDa protein and malate dehydrogenase. Analysis of the *M*. *mycetomatis* grain proteome at day 3 via Blast2GO revealed enrichment of GO terms associated with biological process (catabolic process, macromolecule localization, oxidation-reduction process and response to stress), molecular function (hydrolase activity, protein binding and drug binding), cellular component (non-membrane-bounded organelle, ribonucleoprotein complex and cell periphery) and enzyme categories (Hydrolases, Transferases and Oxidoreductases) (**Fig 4, S1 Table)**.

Out of the 997 *G*. *mellonella* proteins, 488 SSDA proteins were identified in the 3 day grain proteome relative to the 1 day grain proteome **(S6 Table).** Grains from larvae infected with *M*. *mycetomatis* for 3 days [which were compared to grains isolated from 1 day infected larvae] showed an increase in *G*. *mellonella* proteins such as AGAP010145-PA (39 fold), ATP synthase subunit alpha (13 fold), transferrin (12 fold), hdd11 (4 fold), hemicentin-like protein 2 (3 fold), as well as a range of proteins associated with the ribosomal (ribosomal protein L7, 40S ribosomal protein S16, 60S ribosomal protein L13a, Ribosomal protein S27A, L35, S12, L-37, **(S6A Table).** A reduction in the abundance of antimicrobial peptides cobatoxin-like protein (14 fold), cecropin-D-like peptide (5 fold), gloverin (4 fold) and anionic antimicrobial peptide 2 (3 fold) **(S6B Table)** was seen in the 3 day old grain compared to the 1 day old grain.

#### Hemolymph

In the hemolymph, in total, 26 *M*. *mycetomatis* proteins and 3217 *G*. *mellonella* peptides representing 330 proteins with two or more peptides were identified. The most abundant *M*. *mycetomatis* proteins were Heat shock 70 kDa protein, Alpha-1,4 glucan phosphorylase, 3-dehydroshikimate dehydratase, Trans-aconitate 2-methyltransferase, Small COPII coat GTPase SAR1, Tricalbin-3, 60S ribosomal protein L27, Clathrin heavy chain, Succinyl-CoA:3-ketoacid-coenzyme A transferase and malate dehydrogenase **(S5 Table)**.

In terms of *G*. *mellonella* proteins 114 of the *G*. *mellonella* proteins at day 3 were SSDA as compared to hemolymph of non-infected larvae **(Fig 5B, S7 Table)**. These included Hdd11 (337 fold), transgelin (223 fold), heat shock-like protein (115 fold), Hdd1 (30 fold), glutathione-s-transferase-like protein (25 fold), hemicentin (24 fold), prophenoloxidase activating factor 3 (14 fold) and superoxide dismutase (9 fold), **S7A Table.** Proteins decreased at day 3 as compared to day 0 hemolymph were apolipophorin (33 fold), hexamerin (10 fold), cationic peptide CP8 (4 fold) and 27 kDa hemolymph protein (3.5 fold) (**S7B Table, S8 Table**).

### Proteome at 7 days after inoculation

#### Grains

In the grain proteome, a total of 4746 peptides were detected representing 18 *G*. *mellonella* proteins and 48 *M*. *mycetomatis* proteins. The identified *M*. *mycetomatis* proteins included T-complex protein 1 subunit gamma, Putative voltage-gated potassium channel subunit beta, peroxisomal hydratase-dehydrogenase-epimerase, Ketol-acid reductoisomerase, mitochondrial and Phosphoenolpyruvate carboxykinase. Interrogation of the 3 day *M*. *mycetomatis* grain proteome via Blast2GO revealed enrichment of GO terms associated with biological process (cellular component organization, establishment of localization, cellular component biogenesis, regulation of cellular process), molecular function (heterocyclic compound binding, organic cyclic compound binding, ion binding, protein binding), cellular component (endomembrane system, proteasome regulatory particle, supramolecular polymer) and enzyme categories (Hydrolases) (**Fig 4)**.

In *G*. *mellonella*, a total of 96 SSDA proteins were identified in the day 7 grain proteome relative to the 3 day grain proteome **(S9 Table).** By 7 days there was a significant decrease in the number of identified total proteins from grains as compared to the 1 day grain proteome. For example, lysozyme-like protein 1 (9 fold), hemolymph proteinase 16 (9 fold) and hemicentin-like protein 2 (9 fold) were increased in abundance **(S9A Table)** while Heat shock protein 25.4 (358 fold), 27 kDa hemolymph protein (76 fold), apolipophorin (75 fold), hemolin (29 fold) and hdd11 (9 fold) were decreased in abundance within 7 day grain samples, **(S9B Table).**

#### Hemolymph

In the hemolymph, in total 3217 *G*. *mellonella* peptides representing 330 proteins with two or more peptides and 36 *M*. *mycetomatis* proteins were identified. The most abundant *M*. *mycetomatis* proteins identified were Vegetative incompatibility protein HET-E-1, actin, Long-chain-fatty-acid—CoA ligase 1, Heat shock 70 kDa protein, Chromodomain helicase hrp3, alpha-tubulin, Dehydrodolichyl diphosphate synthase complex subunit NUS1, Peroxisomal long-chain fatty acid import protein 2, Catechol 1,2-dioxygenase, GTP-binding protein ypt1, Ribosomal protein, Conidiation-specific protein 6, Ras-related protein YPTC6, and Superoxide dismutase 1 copper chaperone.

Out of the 330 *G*. *mellonella* proteins identified, 154 of the *G*. *mellonella* proteins were SSDA when compared to non-infected larvae. At this time point proteins such as Hdd11 (533 fold), heat shock-like protein (250 fold), hemicentin (54 fold), hemolin (44 fold), thioredoxin (41 fold), hemicentin-like protein 1 (37 fold), glutathione-S-transferase-like protein (21 fold), cecropin-A (12 fold), 6tox (3 fold), ferritin (3 fold) and apolipoprotein D-like Protein (3 fold) were increased in abundance (**Fig 5C and S10A Table)**. At the same time point, hexamerin (64 fold), putative hydroxypyruvate isomerase (50 fold), apolipophorin (18 fold), 27 kDa hemolymph protein (9 fold), cationic peptide CP8 (6 fold) and beta-glucan binding protein (3 fold) were all decreased in abundance relative to the 0 day proteome (**S10B Table)**. A range of these proteins play an important role in the antimicrobial response and immune-regulation.

## Discussion

Here, *M*. *mycetomatis* grain formation in *G*. *mellonella* larvae over time was followed by utilising a label-free proteomics approach. Three time points were studied: day 1, day 3 and day 7 after fungal inoculation. On all time points, both *M*. *mycetomatis* as well as *G*. *mellonella* proteins were identified. However, the total number of *M*. *mycetomatis* proteins was lower than expected, possibly due to the high abundance of *G*. *mellonella* proteins relative to *M*. *mycetomatis* proteins present in the grain samples. Furthermore, proteins previously demonstrated to be part of the grain cement material, such as the translationally controlled tumour protein were not identified [9]. Other proteins previously demonstrated to be present in human *M*. *mycetomatis* grains such as fructose biphosphate aldolase (Fba1) were identified in our experiment [8]. The amorphous cement-material present in the grain could hamper the recovery of all protein sequences, furthermore the high frequency of *G*. *mellonella* proteins in grain samples, has possibly over shadowed the majority of the *M*. *mycetomatis* proteins as a higher number of proteins would be expected from this complex sample. Furthermore, since grain formation is the result of the interplay between host and pathogen it could also be that differences can occur when grain formation is studied in different hosts. Proteins identified in human grains might not be present in grains formed in the *G*. *mellonella* host, only a proteomic comparison between grains formed in different host can solve this question. Although it is likely that not all proteins were recovered, from the proteins which were recovered we could form an idea of the processes involved in mycetoma grain formation.

### Step 1: Recognition of pathogen and host

**Fig 6:**
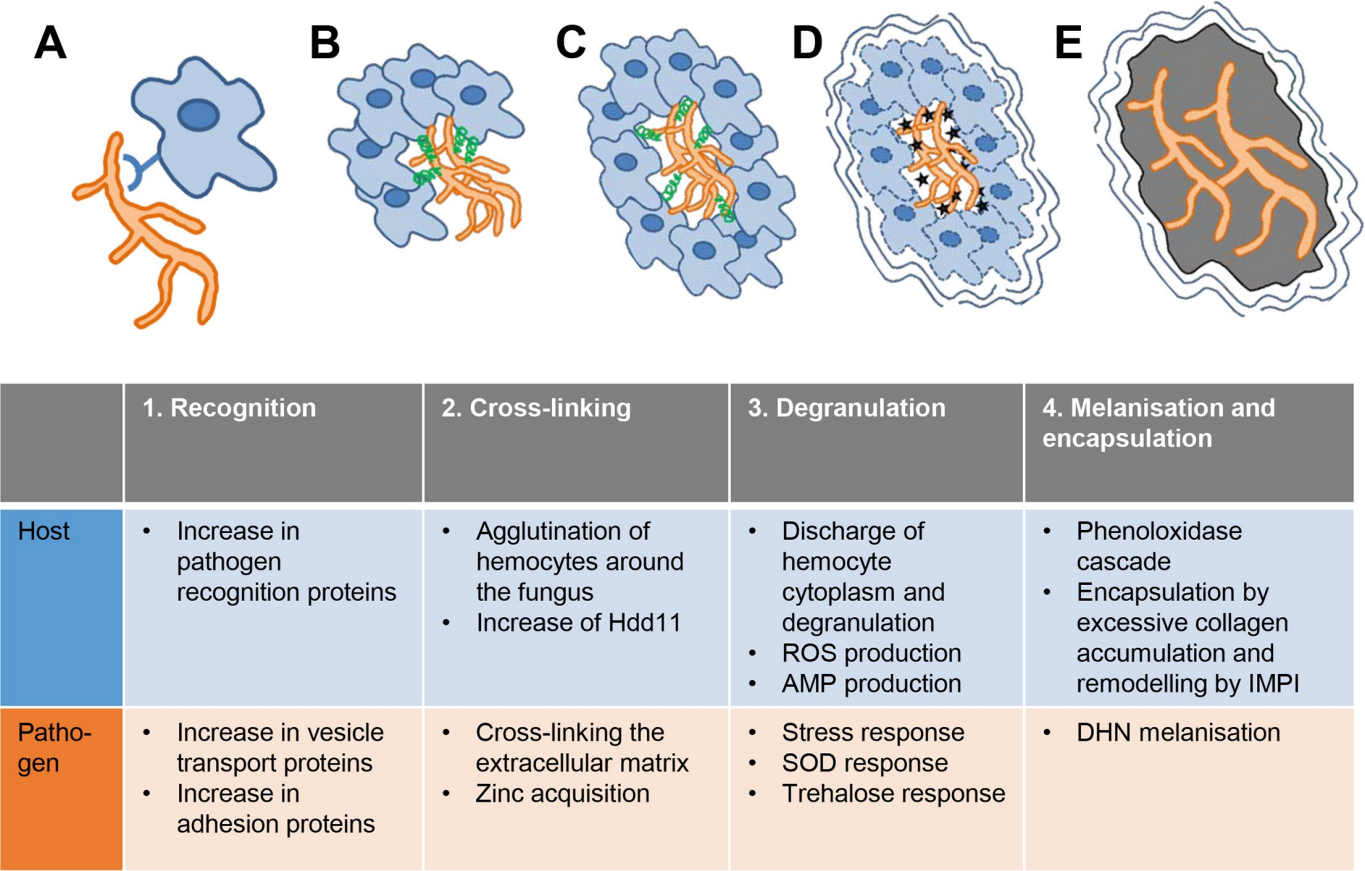
Model of grain formation over time with the most important processes of host and pathogen. A. *M*. *mycetomatis* is recognized by the *G*. *mellonella* host via pathogen recognition proteins. *M*. *mycetomatis* increases vesicle transport and adhesion proteins are displayed on the surface attaching itself to the host. B. Hemocytes will agglutinate around the fungal hyphae and Hdd11 production is increased resulting in crosslinking of the hemocytes and attaching to the fungus. The fungus itself will secrete Asp f2, a zincophore to acquire zinc and to cross link the extracellular matrix. C. Hemocyte cytoplasm will be discharge and degranulation occurs which elevates ROS production and the secretion of AMPs at the granule. The fungus will react by producing SOD and trehalose. D. Melanin will be produced by the host and by the fungus and a capsule is formed surrounding the grain. E. In the last stage, no hemocytes are found within the grain, they are all lysed and the extracellular matrix is completely melanised.

A specific feature of the innate immune system of insects is nodulation where multicellular hemocytic aggregates are formed that entrap a large number of micro-organisms [40] (Fig 6). The process of nodulation starts within the first few minutes after hemolymph penetration [41] with the increase of proteins associated with tissue disruption due to fungal proliferation and hyphal formation such as muscle 20 like protein, tropomyosin 2, paramyosin, alpha-tubulin, troponin T, calreticulin, CALNUC, actin 3 and calponin. These proteins were increased in abundance between +2.57 to +190.84 times in the hemolymph of 1 day *M*. *mycetomatis* infected *G*. *mellonella* larvae as described previously for *C*. *albicans* but not *A*. *fumigatus* infection of larvae [19, 20]. Also proteins able to recognize fungal PAMPs were highly abundant, such as peptidoglycan recognition like proteins which also bind β-glucan (PG-RPs)[42], the opsonin lipopolysaccharide binding protein and hemolin. PG-RP LB and PG-RP B were found to be increased in both the grain (+43.49 and +28.51 fold) as well as in hemolymph (+16.91 and +12.21 fold) 1 day post infection with *M*. *mycetomatis*, while other β-glucan recognition proteins, such as β-glucan recognition protein and apolipophorin [29] were decreased in abundance, as also found in other fungal infections [19, 20]. Opsonin lipopolysaccharide binding protein was decreased in abundance (-4.74 fold) in hemolymph and immunoglobulin superfamily member hemolin was increased in abundance in the 1 day old grain (+19.04 fold) while a decreased abundance was noted in the 7 day old grain (-28.51 fold).

In order to form or reshape the forming extracellular matrix within the *G*. *mellonella* nodule, *M*. *mycetomatis* building blocks are transported through the cell wall into the extracellular space. In fungal cells, this follows an endoplasmic reticulum-*trans-*Golgi-plasma membrane route, where a coordinated network of vesicle transport promotes vesicular fusion with the plasma membrane and the release of the cargo to the extracellular space [43]. In both the grain and the hemolymph, many *M*. *mycetomatis* proteins in vesicle transport were found. Of these, Sly1p, tricalbin-3 and small COPII coat GTPase SAR1 are found in the secretory vesicles in the endoplasmic reticulum [44, 45], while sortilin and clathrin are mainly found in the Golgi apparatus [46–48]. In *C*. *albicans* biofilms, the vesicle composition showed a high degree of similarity with the matrix protein and polysaccharide contents, suggesting that extracellular vesicles may be a major source of matrix material [43, 49]. Furthermore, proteins able to bind extracellular components such as laminin (GAPDH, EF-2, Eno1, Fba1, transaldolase) [50, 51], collagen (GAPDH)[52], plasminogen (GAPDH, Eno1, Fba1, Asp f2)[53], fibrinogen (beta-glucosidase) [54] and fibronectin (GAPDH, Ef2, transketolase and 6-phosphogluconate dehydrogenase) [50, 51] were found in the 1 day grain proteome. The presence of Fba1 in the mycetoma grain was confirmed by immunohistochemistry [8]. Laminin and collagen are ancient proteins and known constituents of basal membranes and these *M*. *mycetomatis* proteins could play a role in the encapsulation of the grain noted by day 3 of the grain formation.

### Step 2: Cross-linking of hemocytes and pathogens to form a grain

After recognizing the pathogen, *G*. *mellonella* hemocytes begin to agglutinate around the pathogen forming an overlapping sheath around it. One of the proteins which plays an essential role in the crosslinking of hemocytes and pathogens during nodule formation is Noduler, or the *G*. *mellonella* homologue Hdd11 [55]. It binds to yeast β-1,3-glucan and traps micro-organisms and hemocytes into the nodule. In *M*. *mycetomatis* infected *G*. *mellonella* larvae, Hdd11 was increased +147.04 fold in the grain and +47.19 fold in *M*. *mycetomatis* infected larval hemolymph. The increase of Hdd11 in hemolymph was comparable to the +49.4 fold increase of this protein found in hemolymph of *C*. *albicans* infected *G*. *mellonella* larvae and was much higher than the +3.66 fold increase observed in *A*. *fumigatus* infected *G*. *mellonella* larvae [19, 20]. The Hdd11 concentrations further increased in 3 day old grains compared to 1 day old grains (+4.07 fold) and then decreased in 7 day old grains (-8.68 fold, when compared to 3 day grain proteome).

Next to Hdd11, other immune-related proteins were also increased in the *M*. *mycetomatis* grain at 1 day after infection. These included Hdd1 (+26.04 fold), Hdd23 (+21.03 fold) and Hdd1-like protein (+18.62 fold) which have been identified as playing an important role in the nodulation response. Also in hemolymph Hdd1 was increased by +26.71 fold during *M*. *mycetomatis* infection. This increase was higher than previously reported for *C*. *albicans* infected *G*. *mellonella* larvae (+13.5 fold increase) and *A*. *fumigatus* infected *G*. *mellonella* larvae (+3.79 fold) at the same time point [19, 20].

Next to *G*. *mellonella*, *M*. *mycetomatis* itself also seems to play a role in the cross-linking of the extracellular matrix. In the day 1 grain proteome the Asp2f homologue was found. This protein and its *Candida albicans* homologue Pra1 are secreted from the fungal cell to form a complex with extracellular zinc and are recruited back to the fungal cell [56–58]. Once near the *C*. *albicans* cell, the zinc bound by Pra1 is cross-linked to the amyloid regions of the aspartic proteinase Sap6, resulting in large fungal aggregates with elevated zinc concentrations similar to biofilms [59]. Although a homologue of Sap6 was not identified in our experiments, another amylolytic protein was found 3 days post infection, namely alpha/beta-glucosidase agdC [60]. Strikingly, elevated levels of zinc were noted within mycetoma grains in humans [10, 61] which could indicate that a similar cross-linking activity of Asp2f also takes place in the production of the cement material noted in the *M*. *mycetomatis* grains.

### Step 3: Degranulation of *G*. *mellonella* hemocytes and the response to reactive oxygen species (ROS)

Aggregation of granular cells followed by degranulation is typical for the *G*. *mellonella* nodule formation and leads to the accumulation of coagulogen around the fungus [41]. Discharge of hemocyte cytoplasm and granule contents is followed by melanisation. Degranulation of the granular cells induces the synthesis of nitric oxide by NO synthase and ROS. During encapsulation usually a significant increase in ROS and a decrease in enzymatic antioxidant activities such as superoxide dismutases have been noted. In *G*. *mellonella* infected with *M*. *mycetomatis* an increase in the superoxide dismutase activity (+115.62 fold) and peroxidase (+7.92 fold) is noted in 24 h old grains.

Next to ROS, antimicrobial peptides were also found within the forming grain. Antimicrobial proteins such as cecropin-D (+175.74 fold), gloverin (+114.8 fold), 6tox (+47.46 fold), lysozyme (+17.99 fold), gloverin-like protein (+10.84 fold) and anionic antimicrobial peptide 2 (+1.89 fold) were increased in 24 h grains relative to control hemolymph, while cationic peptide CP8 (-5.44 fold) was decreased in abundance at this time point. Lysozyme was found highly enriched within *M*. *mycetomatis* grains but absent in hemolymph which may confirm (along with other proteins e.g. apolipophorin and β-glucan recognition proteins) that certain proteins are shuttled from the hemolymph to the site of infection to act directly at the site of infection. At the site of infection, lysozyme binds to the fungal cell surface (i.e. membrane or cell wall) and will cause osmotic imbalance and cell death as reported in *C*. *albicans* [62, 63]. Like lysozyme, the α-helical cecropins and pro-peptide gloverins also target the fungal cell surface and induce apoptosis [64–66]. Another class of antimicrobial peptides of *G*. *mellonella* which is highly active against both yeasts and filamentous fungi [67] is the moricins. Moricins are secreted as pro-peptides and are activated via proteolysis to increase the permeability of bacterial and fungal membranes. *G*. *mellonella* has seven moricin-like peptides in its transcriptome and none of them was detected after *M*. *mycetomatis* infection. In contrast, moricin was found to be increased by +20.6 during *C*. *albicans* infection and moricin C1 was increased by +15.84 fold in *A*. *fumigatus* infected larvae relative to control larvae [19, 20]. This may indicate that *M*. *mycetomatis* can manipulate the host response to suppress the expression of moricin AMPs as they were also not detected in *M*. *mycetomatis* grains.

The ROS and AMPs generated by *G*. *mellonella* will evoke a stress response in *M*. *mycetomatis*. It is therefore to be expected that a range of *M*. *mycetomatis* proteins would be detected in the early grain phase which could protect the fungal cells. Indeed in the 1 day old grains, SOD, trehalose-phosphatase and trehalase were found to be present, which have been implicated in the protection against stress in other fungal species [68]. During stress, trehalose can interact with proteins and phospholipids to protect membrane structures and prevent protein denaturation [69]. Furthermore, trehalose can scavenge free radicals under oxidative stress conditions and can protect against host defenses [69, 70]. In *C*. *albicans*, trehalose levels were also found in the earliest phases of biofilm formation, while they were decreased in mature biofilms [71].

Over time, as the infection progresses in *G*. *mellonella*, the abundance of AMPs within the grain proteome decreased. Within the 72 h grain proteome a decreased abundance of AMPs such as cecropin-D (-4.55 fold), gloverin (-3.97 fold), cationic peptide CP8 (-3.39 fold), anionic antimicrobial peptide 2 (-2.92 fold), cobatoxin-like protein (-14.39 fold) relative to 24 h grains was noted. This was also observed at 7 day relative to 72 h grains with anionic antimicrobial peptide 2 (-53.47 fold), cecropin-D-like peptide (-11.52 fold) all decreased in abundance, with the exception of lysozyme-like protein 1 (+9.35 fold) which was increased in abundance. Also a difference in the type of *M*. *mycetomatis* proteins present was noted. In the more mature grain, where the nutrients might be more depleted a metabolic conversion seems to occur in the 7 day old grains, as at this time point isocitrate lyase and phosphoenolpyruvate carboxykinase were present. These enzymes are normally down-regulated in the presence of glucose, which suggests that at this time in grain formation the fungal cells are surviving on alternative carbon sources. They were also linked to the persister cell phenotype in *C*. *albicans* biofilms, where they represent a more dormant state of the fungus [72].

#### Step 4: Melanisation of the grain

During degranulation of the hemocytes prophenoloxidase (proPO) is released. This starts the melanisation process of the *G*. *mellonella* nodule. Indeed, 1 day after *M*. *mycetomatis* inoculation, members of the phenoloxidase cascade [prophenoloxidase activating enzyme 3 (+47.28 fold), prophenoloxidase subunit 2 (+11.51 fold)] were increased in abundance in the grain as well as in hemolymph (prophenoloxidase activating enzyme 3 (+10.72 fold); prophenoloxidase activating protease 1 (+5.56 fold)). This elevation was stronger compared to what was reported for *C*. *albicans* (+1.8 fold) and *A*. *fumigatus* (+2.44 fold) [19, 20]. Melanisation kills pathogens by restricting nutrition uptake from the surroundings due to the formation of a thick surrounding layer, very similar to the mammalian complement cascade [73–75]. However, for many pathogens, melanisation is also a defense mechanism present in their own protective reponse arsenal. In the *M*. *mycetomatis* grain found in human, DHN-melanin is present and the cement material itself is melanised [7]. In *A*. *fumigatus*, the enzymes responsible for DHN-melanin production are localised in endosomes [76] and these endosomes are transported to the cell wall. The last steps of melanisation occur at the cell wall. With the high abundance of *M*. *mycetomatis* proteins involved vesicle transport and the natural melanisation of nodules within *G*. *mellonella* larvae, it is highly likely that in *G*. *mellonella*, the grain is melanised via both the *G*. *mellonella* proPO pathway and the *M*. *mycetomatis* DHN-melanin pathway. In *G*. *mellonella* high oxidative stress is created during the production of melanin and its intermediates. This highly oxidative environment is lethal for most pathogens but also to the *G*. *mellonella* hemocytes itself [73–75]. To prevent excessive tissue damage, the phenoloxidase cascade is highly regulated. It can be activated by apolipophorin III and inhibited by lyzozyme, anionic peptide-2 and serpins in *G*. *mellonella* [77]. Early in the grain formation a decrease of apolipophorins (-4.71 and -3.43 fold) and Serpin 5 was found inside the grain (-1.83 fold) indicating that early in the grain formation melanisation was activated. The decrease in apolipphorins further increased by day 3 (-32.99 fold in hemolymph) and day 7 (-75.17 fold in the grain and -17.63 fold in hemolymph).

After melanisation, the encapsulation process is often terminated by forming a basement membrane like layer around the capsule periphery. This was also noted at day 3 after grain formation indicating that the formation of the grain took 3 days in *G*. *mellonella* larvae [41]. In many organisms, encapsulation occurs due to excessive collagen accumulation around the micro-organism. In the *M*. *mycetomatis* grain found in humans, thick collagen capsules are present which are thought to be produced by the action of matrix metalloproteinases (MMPs) and tissue inhibitors of matrix metalloproteases (TIMPs). Active MMP-9 was present in the serum of mycetoma patients and found to be expressed in the tissues surrounding the grain [78, 79]. Genetic differences in TIMP-1 were associated with mycetoma development [79]. Here, we found that insect metalloproteinase inhibitor (IMPI) was increased by +9.30 fold in *M*. *mycetomatis* infected *G*. *mellonella* larvae compared to non-infected larvae and could play a similar role as TIMP in the formation of a capsule surrounding the *M*. *mycetomatis* grain.

## In conclusion

In this study we have used a proteomic approach to unravel the processes leading to *M*. *mycetomatis* grain formation in *G*. *mellonella* larvae (Fig 6). Our data indicate that grain formation occurs in 4 steps and each of these steps could potentially be inhibited to prevent grain formation. On the pathogen-side inhibiting vesicle transport, zinc-acquisition, trehalose response or melanisation could be novel ways to interfere with grain synthesis. Indeed, sortins have been known to inhibit vesicle transport in *C*. *albicans* and act synergistically with fluconazole [80], validamycin A has been known to inhibit the fungal trehalose pathway in *Rhizoctonia solani* [81] and *M*. *mycetomatis* melanisation was inhibited by tricyclazole [7]. Also interfering with host processes leading to grain formation might open novel ways to treat mycetoma. Inhibiting the capsule formation by MMP inhibitors such as doxycycline, minocycline, incyclinide or anti-MMP antibodies could make the grain more accessible to antifungal agents [82]. Interfering with the immune system could also be beneficial. Diclofenac can prevent the formation of brain nodules after infection with *Listeria monocytogeneses*. Interestingly, diclofenac was also able to cure mycetoma in a patient. Thus, the insights obtained in this study in the *M*. *mycetomatis* grain formation in *G*. *mellonella* larvae can in the future be used to develop novel therapeutic strategies for mycetoma.

## Supporting information

S1 Table *M*. *mycetomatis* proteins in grains isolated from larvae infected with *M*. *mycetomatis*. In this table proteins identified 1 day, 3 days and 7 days after fungal inoculations are depicted.
(XLSX)

S2 Table *G*. *mellonella* proteins increased (S2A Table) or decreased (S2B Table) in abundance in grains isolated from larvae infected with *M*. *mycetomatis* for 24 hour as compared to control larval hemolymph proteome.
(XLSX)

S3 Table *M*. *mycetomatis* proteins in hemolymph isolated from larvae infected with *M*. *mycetomatis*. (XLSX)

S4 Table *G*. *mellonella* proteins increased (S4A Table) or decreased (S4B Table) in abundance in 24 hour *M*. *mycetomatis* infected *G*. *mellonella* larval hemolymph as compared to 0 hour hemolymph.
(XLSX)

S5 Table Enrichment for GO terms (Biological Process [A], Molecular Function [B] and Cellular Component [C]) from the total SSDA **hemolymph** proteins from *G*. *mellonella* larvae infected with *M*. *mycetomatis* (24 hour relative to 0 hour hemolymph proteome).
(XLSX)

S6 Table *G*. *mellonella* proteins increased (S6A Table) or decreased (S6B Table) in abundance in grains isolated from larvae infected with *M*. *mycetomatis* for 72 hour as compared to grains extracted from larvae infected with *M*. *mycetomatis* for 24 hour. (XLSX)

S7 Table *G*. *mellonella* proteins increased (S7A Table) or decreased (S7B Table) in abundance in 72 hour *M*. *mycetomatis* infected *G*. *mellonella* larval hemolymph as compared to 0 hour hemolymph. (XLSX)

S8 Table Enrichment for GO terms (Biological Process [A], Molecular Function [B] and Cellular Component [C]) from the total SSDA hemolymph proteins from *G*. *mellonella* larvae infected with *M*. *mycetomatis* (72 hour relative to 0 hour hemolymph proteome).
(XLSX)

S9 Table *G*. *mellonella* proteins increased (S9A Table) or decreased (S9B Table) in abundance in grains isolated from larvae infected with *M*. *mycetomatis* for 7 days as compared to grains extracted from larvae infected with *M*. *mycetomatis* for 24 hour. (XLSX)

S10 Table *G*. *mellonella* proteins increased (S10A Table) or decreased (S10B Table) in abundance in 7 days *M*. *mycetomatis* infected *G*. *mellonella* larval hemolymph as compared to 0 hour hemolymph. (XLSX)

S11 Table Enrichment for GO terms (Biological Process [A], Molecular Function [B] and Cellular Component [C]) from the total SSDA hemolymph proteins from *G*. *mellonella* larvae infected with *M*. *mycetomatis* (7 day relative to 0 hour hemolymph proteome)
(XLSX)
